# Effect of Stacking Sequence and Sodium Bicarbonate Treatment on Quasi-Static and Dynamic Mechanical Properties of Flax/Jute Epoxy-Based Composites

**DOI:** 10.3390/ma12091363

**Published:** 2019-04-26

**Authors:** Vincenzo Fiore, Luigi Calabrese

**Affiliations:** 1 Department of Engineering, University of Palermo, Viale delle Scienze, 90128 Palermo, Italy; 2 Department of Engineering, University of Messina, Contrada Di Dio, 98158 Sant’Agata, Messina, Italy; lcalabrese@unime.it

**Keywords:** green composites, sodium bicarbonate treatment, hybridization, mechanical characterization

## Abstract

The present paper deals with the investigation of quasi-static and dynamic mechanical response of epoxy-based composites reinforced with flax and/or jute plain weave fabrics. In order to evaluate the influence of the stacking sequence, two monolithic laminates reinforced with flax or jute fibers and two hybrid flax/jute laminates were manufactured through the vacuum infusion technique. Furthermore, an eco-friendly and cost-effective surface treatment based on fiber soaking in a sodium bicarbonate solution was employed to improve the fiber-matrix adhesion. The mechanical characterization (by means of quasi-static flexural, dynamic mechanical thermal analysis and Charpy impact tests) allowed to evidence that the sodium bicarbonate treatment leads to noticeable improvement of the mechanical performances of flax reinforced composites, whereas jute composites experience a slight decrease of their mechanical properties. Overall, the hybridization allows to achieve intermediate mechanical properties among those of monolithic composites. Furthermore, the coupled action of hybridization and surface treatment does not lead to a beneficial and reliable effect on the mechanical response of the resulting composites.

## 1. Introduction

The production of composite materials from bio sources have gained increased interest over the last decades with the aim to reduce the environmental pollution and to create a closed loop for products’ lifecycle [1,2]. In this context, lignocellulosic fibers find, nowadays, established applications mainly in the building and automotive fields as reinforcement of polymeric-based materials thanks to their specific mechanical properties, low density, low raw material cost, low-cost production and processing, large availability, lightweight, biodegradability, and recyclability [3,4]. Furthermore, in contrast to synthetic fibers, such as glass, carbon, and Kevlar, these fibers are renewable and have a CO_2_-neutral life cycle. 

In spite of this, natural fibers also present several drawbacks that make their use feasible only in non-structural or semi-structural applications, thus limiting the large-scale industrial adoption of natural fiber composites [5]. In particular, besides their inherently variability in mechanical properties and high tendency to moisture absorption, the main weaknesses of these fibers are represented by lower mechanical properties than the synthetic counterparts and poor adhesion with several different polymeric matrices [6,7].

In order to overcome these issues thus obtaining natural fiber reinforced composites with tailored mechanical performances, possible solutions are represented by surface treatments of natural fibers and/or their hybridization (e.g., with synthetic or dissimilar lignocellulosic fibers).

With regard to fiber treatments, both physical [8,9,10] and chemical methods [11] have been employed with the aim to improve the compatibility between hydrophilic natural fibers and hydrophobic polymeric matrices [12]. Among chemical approaches, the most widely adopted is the alkali treatment also called mercerization. Several papers are available in the literature concerning the effects of this treatment on the composites properties as a function of solution concentration, temperature and immersion time [13,14,15,16]. 

Even if chemical treatments lead to noticeable improvement of the fiber-matrix compatibility, they are often expensive and environmentally harmful due to the large amount of hazardous chemicals involved, thus making them industrially less attractive. On this basis, an eco-friendly and cost-effective treatment based on the soaking of natural fibers in a sodium bicarbonate solution was carried out in our previous paper [17] with the aim of improving the compatibility of short sisal fibers to an epoxy matrix. Sodium bicarbonate is not environmentally dangerous or harmful for human health (i.e., it is frequently used as antacid for medical purposes, as leavening agent to bake cakes and bread and in the manufacture of sodium carbonate—Na_2_CO_3_). Furthermore, the very low cost of the raw material makes its use promising for large-scale industrial technologies, leading this surface treatment approach potentially reliable and effective in the industrial field.

Thanks to the results achieved in this first paper, other researchers focused their attention during the last years on the effect of this new method on the adhesion between epoxy and polyester matrices with coir fibers [18,19], epoxy with flax fibers [20], and poly-lactic acid with sisal fibers [21], thus confirming the suitability and versatility of the proposed treatment. 

Another approach widely used to properly tailor the mechanical and physical properties of natural fiber reinforced composites is the hybridization of these natural fibers with stronger and more aging resistant synthetic fibers such as glass [22,23,24,25] carbon [26,27,28,29,30,31,32] and Kevlar [33,34] or mineral fibers, such as basalt [35,36,37,38,39,40]. On the other hand, few literature references are available until now on hybrid composites reinforced with different natural fibers [41,42,43]. For instance, Karaduman et al. [41] evaluated the effect of stacking sequence on the mechanical properties of hybrid flax/jute fibers reinforced thermoplastic composites using polypropylene and maleic anhydride grafted as matrix materials. Mohan and Rajmohan [42] evaluated the mechanical properties of hybrid banana/flax/jute composites as a function both of the stacking sequence and of the weight content of multi-walled carbon nanotubes. Wear and dynamic mechanical behavior of jute/hemp/flax-reinforced composites were analyzed by Chaudhary et al. [43] with the aim of using them in tribological applications.

However, a study aimed to assess the relationship between the mechanical performances in quasi-static and dynamic conditions of monolithic and hybrid natural fiber composites and how they can be influenced by an innovative fiber surface treatment is particularly needed in order to deepen the knowledge of this class of materials in specific conditions.

In such a context, the present paper deals with the investigation of hybridization and fiber treatment on the mechanical properties of natural fiber reinforced composites. In more detail, two woven fabrics having the same architecture and similar areal weight were used to evaluate how the hybridization of two natural fibers such as flax and jute having apparently similar mechanical properties can influence the mechanical response of the resulting composites. Moreover, a promising eco-friendly treatment was employed with the aim of improving the mechanical properties of the composites, thus extending their use to a wide range of applications.

## 2. Materials and Methods 

Eight composite laminates (i.e., four reinforced by fabrics as received and four with treated fabrics) with dimension of 300 mm × 300 mm were manufactured, in the composite materials laboratories of University of Palermo, through vacuum assisted resin infusion [44,45], a close mold technique where dry plies are placed and compacted by a vacuum bag. In particular, the dry fabrics contained in the mold is then impregnated by resin, which flows through, mainly driven by vacuum [46]. All the laminates were cured at 25 °C for 24 h and post-cured at 50 °C for 8 h. A two-stage vacuum pump was used to create maximum vacuum equal to 0.1 atm (absolute).

Two twill weave woven fabrics were used in the manufacturing of the laminates: i.e., jute, supplied by Composites Evolution (Chesterfield, UK), and flax supplied by Lineo (Valliquerville, France), having areal weight of 400 g/m^2^ and 320 g/m^2^, respectively. An epoxy resin SX8 EVO supplied by Mates Italiana s.r.l. (Segrate-Milano, Italy) mixed with its own amine-based M-type (medium reactivity) hardener (100:30 mix ratio by weight) was used as matrix. With regard to the fiber treatment, both fabrics were immersed in a solution of sodium bicarbonate (10 wt%) for five days at 25 °C. At the end of the soaking, fabrics were washed with distilled water and dried in an oven at 40 °C for 24 h. Afterwards, they were dried at 103 °C for further 24 h in an oven to remove the residual moisture.

Table 1 reports the stacking sequence, nominal thickness, volume contents (i.e., fiber, matrix, and void), and theoretical and experimental densities for each laminate. 

The experimental density *ρ_e_* was measured using a Pycnomatic ATC helium pycnometer (Thermo Electron Corporation, Waltham, MA, US) whereas the theoretical density *ρ_t_* was calculated with the following equation:

(1) $$\rho_{t}=\frac{1}{\left(\frac{W_{f}}{\rho_{f}}\right)+\left(\frac{W_{m}}{\rho_{m}}\right)}$$

where *ρ_m_* and *W_m_* represent the density and the weight content of epoxy matrix, respectively, whereas ρ_f_ and *W_f_* represent the density and the weight content of fiber, respectively.

Void volume fraction (*ν_V_*) of each composite was evaluated by comparing its experimental and theoretical densities as the following:

(2) $$\nu_{V}=\frac{\rho_{t}-\rho_{e}}{\rho_{e}}$$

For the mechanical characterization, specimens were cut from the laminates to their nominal dimensions depending on the specific test, by using a water-cooled diamond blade saw. 

### 2.1. Quasi-Static Flexural Tests 

Three point bending tests were carried out according to ASTM D790 standard on prismatic samples (15 mm × 80 mm) using an electromechanical universal testing machine model Z005 (Zwick-Roell, Ulm, Germany), equipped with a 5 kN load-cell (Zwick-Roell, Ulm, Germany). The support span was set to 64 mm and the crosshead speed to 2 mm/min. Five specimens for each condition and layup were tested.

The flexural stress *σ* and strain *ε* were calculated with the following equations:

*σ* = 3*PL*/2*bd*^2^ (3)

*ε* = 6*Dd*/*L*^2^ (4)

where *P* is the applied load (N), *L* the support span (mm), *b* the sample width (mm), *d* the sample depth (mm), and *D* the midpoint deflection (mm), respectively.

### 2.2. Dynamical Mechanical Thermal Analysis 

Dynamic mechanical thermal tests were performed in tensile mode according to ASTM D 4065 standard, using a dynamic mechanical analyzer model DMA+150 (Metravib, Limonest, France). Three prismatic specimens (3 mm × 46 mm) per laminate were tested from room temperature to 150 °C with heating rate of 3 °C/min, in nitrogen atmosphere. The dynamic displacement and the displacement frequency were set equal to 5 × 10^−6^ m and 1 Hz, respectively.

### 2.3. Charpy Impact Tests

Impact tests were carried out according to EN ISO 179 standard, using a Charpy pendulum model 9050 (CEAST, City, Italy), equipped with a pendulum of potential energy equal to 5 J and impact speed of 3.8 m/s. Five un-notched prismatic specimens (80 mm × 10 mm) of each laminate were tested at room temperature.

### 2.4. Morphological Analysis

Morphological analysis was performed on the fractured surfaces of impact tested specimens by using a Phenom Pro X scanning electron microscope, SEM (Phenom World, Eindhoven, Netherlands). Before analysis, each specimen was sputter-coated with a thin layer of gold to avoid electrostatic charging under the electron beam. The micrographs were obtained at a voltage of 15 kV.

## 3. Results and Discussion

### 3.1. Quasi-Static Flexural Tests

#### 3.1.1. Effect of Surface Treatment

**Figure 1:**
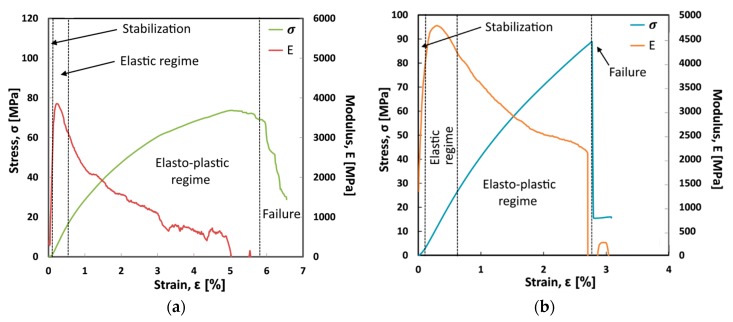
Flexural stress and modulus vs. strain typical curves of (**a**) Flax-AR and (**b**) Jute-AR laminates.

Flexural stress and modulus versus strain typical curves for jute and flax untreated laminates (i.e., Flax-AR and Jute-AR) are shown in Figure 1.

Four regions can be identified:

1. Initially, a stabilization region at very low strain is evident. The stress and flexural modulus are quite low and progressively increase at increasing strain. This region can be ascribed to the tools adjustment and it is not related to a mechanical behavior of the composite laminates;
2. Afterward, the stress-strain curve has a linear trend, indicating a clear elastic regime. A maximum in modulus is reached both for jute and flax laminates with the former stiffer than the latter one;
3. However, at increasing strain, both laminates show a deviation from the linear trend, showing a marked elastic-plastic regime. This behavior is more pronounced for the flax laminate as shown by the progressive reduction of the flexural modulus trend from about 4000 MPa to about 1000 MPa. On the other hand, in the jute sample the elasto-plastic regime is less pronounced and a modulus reduction from about 4700 MPa to about 2500 MPa can be identified. The maximum in stress is reached for both laminates (i.e., equal to about 90 MPa and 75 MPa for jute and flax laminates, respectively);
4. Finally, the sample fracture occurred. In the case of the jute laminate the fracture found is catastrophic with a sudden and abrupt collapse of the load. In the flax laminate the fracture, evidenced stepwise trend with a slight progressive evolution of the damage that falls down progressively up to 30% of the maximum load with a maximum strain of 6.5%.

**Figure 2:**
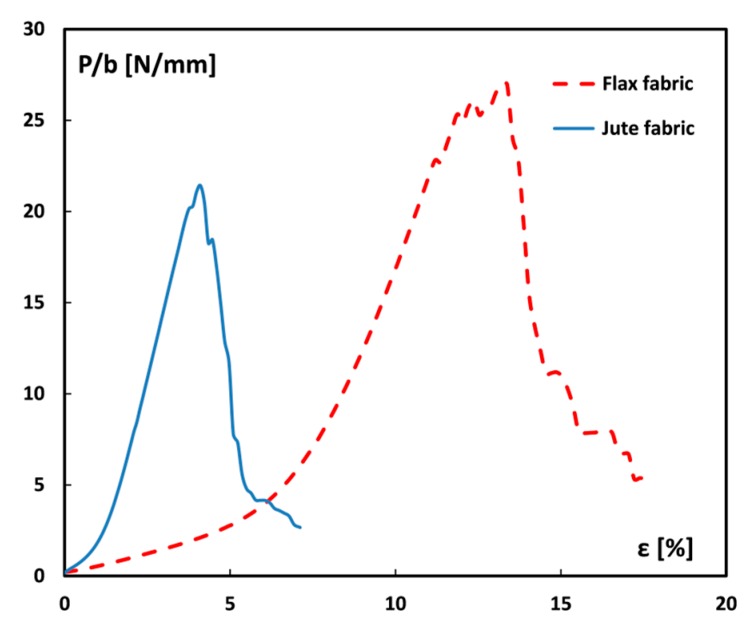
Stress-strain tensile curves of dry flax and jute fabrics.

The comparison of the flexural curves (Figure 1) clearly provides a first indicative discrimination between the laminates. Jute-AR laminate has a predominantly elastic behavior with a brittle and catastrophic fracture. The flexural strength and modulus are equal to 88.8 MPa and 4.78 GPa, respectively, whereas the strain at maximum stress is equal to 2.77%. Conversely, Flax-AR laminate shows a mainly elasto-plastic behavior with a lower strength and stiffness (i.e., 73.7 MPa and 3.85 GPa, respectively) but higher strain at maximum stress (i.e., 5.0%) compared to its counterpart. These results can be ascribed both to the mechanical behavior of dry fabrics and to the fiber adhesion with the epoxy resin used as matrix. In particular, tensile tests on dry fabrics showed that jute fabrics result stiffer and less resistant than flax ones (Figure 2). Furthermore, the elongation at break of flax fabrics is greatly larger than that of jute fabrics.

Consequently, the higher flexural modulus exhibited by the jute laminate can be due to the stiffer behavior of jute fabrics in comparison to flax ones. On the other hand, the jute laminate shows higher flexural strength than the flax one thanks to the better adhesion between jute fibers and epoxy matrix, as will be shown by the morphological analysis. Moreover, the higher deformation at break shown by the flax laminate is mainly due to the greater deformation capability evidenced by flax dry fabrics in comparison to their counterparts.

**Figure 3:**
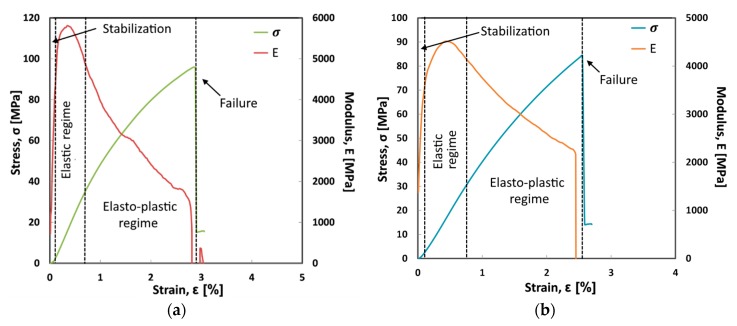
Flexural stress and modulus vs. strain typical curves of (**a**) Flax-T and (**b**) Jute-T laminates.

In order to acquire useful information about the effect of the sodium bicarbonate treatment on the mechanical performances of composite laminates, the stress and flexural modulus trends versus strain for Flax-T and Jute-T batches are reported in Figure 3. 

A significant modification on mechanical performances can be identified comparing Flax-AR and Flax-T laminates, Figure 1a and Figure 3a, respectively. In more detail, the treated flax fibers favor the strengthening and stiffening of the resulting laminates. The strain at failure is significantly reduced coupled to a reduction of the elasto-plastic regime in the stress-strain curve. The Flax-T sample reaches a maximum strength of about 100MPa and an elastic modulus above 5500 MPa. The sodium bicarbonate treatment on flax fibers allowed to obtain a composite laminate with more effective mechanical performances compared to as received flax based one, favoring a strengthening effect on the flax fiber coupled to an improvement of flexural modulus.

On the other hand, Jute-T laminate (Figure 3b), compared with the untreated one (Figure 1b), is characterized by a modulus trend quite similar, indicating that the sodium bicarbonate treatment in the jute fiber does not affect significantly the stiffness of the composite laminate. Moreover, a still brittle fracture behavior can be observed although slight reductions of strength and strain at failure are evident in comparison to Flax-AR laminate. This means that a feeble effect on the adhesion between jute fibers and epoxy resin is achieved through the sodium bicarbonate treatment. Furthermore, the fiber soaking in the NaHCO_3_ solution could favor softening and damaging effects on the jute morphology, leading to the flexural modulus reduction of the resulting composites. A further effect of the sodium bicarbonate treatment consists in the reduction of lignin and hemicellulose amounts in the fiber bulk, thus creating preferential pathways for damage triggering and propagation. This leads to a higher rate of damage growth as a consequence of the lower yarn stiffness [47]. 

**Figure 4:**
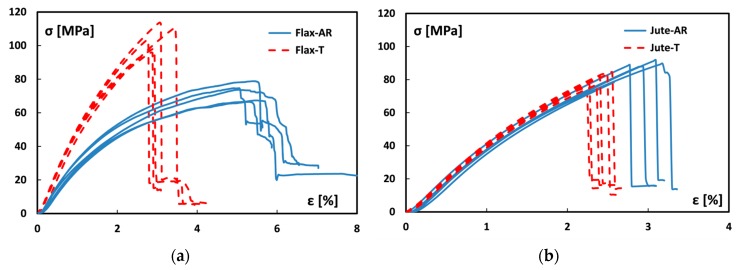
Distribution of stress-strain flexural curves of (**a**) flax and (**b**) jute laminates.

These results clearly point out that fiber-matrix adhesion was greatly enhanced after the sodium bicarbonate treatment for flax fibers whereas, as previously stated, the compatibility between epoxy matrix and jute fibers did not exhibit any improvement. This different behavior is better highlighted by comparing the distribution of stress-strain curves of flax and jute laminates reinforced with raw and treated fabrics (Figure 4). All batches show a quite homogeneous distribution of curve with almost compatible mechanical behavior. In particular, analyzing Figure 4a, all Flax-T samples showed higher mechanical performances compared to Flax-AR laminates. In addition to higher strength and stiffness, Flax-T samples showed a more brittle behavior. The large elasto-plastic deformation, shown by Flax-AR laminates, is significantly reduced and the fracture occurs with an abrupt reduction of the load. On the other hand, no noticeable variation in the slope can be highlighted for jute batches (Figure 4b). Furthermore, premature failures at lower values of stress and strain are observed for all Jute-T laminates in comparison to Jute-AR ones. 

Table 2 summarizes the average values (and related standard deviations) of flexural strength and modulus for monolithic laminates. Moreover, the strain at maximum load was also added in order to better highlight the elasto-plastic behavior of the samples. 

By analyzing the flexural properties of the laminates manufactured using the treated fabrics, it is possible to better quantify how the sodium bicarbonate treatment on natural fibers is able to enhance or to depress stiffness and strength of the resulting composites. In particular, it is evident that the sodium bicarbonate treatment greatly improves the mechanical properties of flax laminates whereas jute laminates do not experience any noticeable variation. In more detail, the average values of flexural strength and modulus of Flax-T laminates (i.e., those reinforced with treated fabric) are +40% and +46% higher than those of Flax-AR laminates (i.e., reinforced with fabric as received), respectively. Consequently, the deformation at break of flax laminates varies from 5.2% to 3.0%, due to the sodium bicarbonate treatment. At the same time, jute laminates do not improve their flexural properties as a consequence of the NaHCO_3_ treatment: i.e., flexural strength decreases from 87.6 MPa to 80.8 MPa, elongation at break from 3.0% to 2.4% whereas flexural modulus remains almost constant (i.e., 4.36 GPa vs. 4.42 GPa). It is also worth noting that the flexural properties of flax laminates are characterized by a standard deviation about twice the one of jute laminates, indicating a larger distribution of data. This experimental evidence can be ascribed to the higher heterogeneity in the morphology of the flax laminate than jute one. As observed by Hamad et al. [48], flax fibers are characterized by irregular fiber cross-section and internal lumen shape, unlike jute fibers that exhibit a homogenous and regular internal lumen structure and a quite circular cross-section shape. This different morphology could induce a lower dispersion in mechanical performances in jute-reinforced composites than in the flax one. The heterogeneity in flax fiber reinforcement could be related to the presence of both elementary and technical fibers (i.e., naturally adhering bundles of elementary flax fibers). Therefore, under mechanical stress, local fractures can occur caused by the elementary fibers that are pulled out both from the matrix or from the technical fiber bundles [49]. The reciprocal adhesion of flax fibers could be locally low, influencing the distribution of the mechanical performances in the composite laminates [50].

#### 3.1.2. Effect of Hybridization 

**Figure 5:**
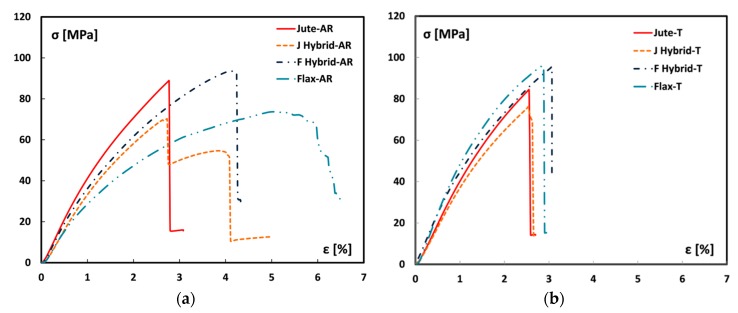
Typical stress vs. strain flexural curves of (**a**) untreated and (**b**) treated laminates.

Further interesting information can be obtained by analyzing the evolution of the mechanical behavior of the laminates as a function of both the stacking sequence hybridization and of the NaHCO_3_ treatment. Figure 5 shows the trend of stress-strain bending curves for hybrid and monolithic laminates reinforced with as received (Figure 5a) and treated vegetable fabrics (Figure 5b). 

By analyzing the bending curves for untreated fiber laminates, it is worth noting that hybrid laminates exhibit an intermediate mechanical behavior between Jute-AR and Flax-AR laminates. F-Hybrid-AR and J-Hybrid-AR show a similar stiffness trend at low strain values even if the hybrid stacking sequence with external jute fibers (i.e., J-Hybrid-AR) evidences a slight lower stiffness compared to F-Hybrid-AR sample. Furthermore, J-Hybrid-AR sample shows a lower strain at failure compared to its hybrid counterpart (i.e., F-Hybrid-AR). This is due to the brittle behavior of the jute fibers which, being placed as external lamina, significantly influence the flexural behavior of the composite laminate itself, thus leading to a premature first play failure (FPF). It is also noteworthy that J-Hybrid-AR laminates exhibit a residual strength, identifiable by the stress step at about 50 MPa, due to the second play failure (SPF) related to the internal flax fibers, which being more flexible fibers allow to suffer deformation states imposed during bending test postponing a complete collapse of the composite laminate. The F hybrid-AR laminate shows only a clear FPF due to tensile fracture of the external flax lamina. Afterward, the internal jute lamina is not able to suffer the residual stress and an abrupt stress reduction at FPF strain takes place. This hybridized stacking sequence represents an effective design compromise, since it maximizes the stress transfer among laminas without implying premature fractures at low stress and strain levels. Indeed, F hybrid-AR sample shows acceptable strain at failure (i.e., about 4%) and very high maximum stress which is also higher than Jute-AR laminate. 

A significantly different behavior is observed by analyzing the treated laminates. The sodium bicarbonate treatment on the vegetable fibers has led to similar mechanical behavior of the two types of fibers. Flax-T and Jute-T laminates have a quite similar stress-strain trend. This implies that also hybrid composite laminates show a significant compatible mechanical behavior. All laminates have a maximum stress of about 80–100 MPa and a strain at failure of about 2.7–3.2%. Nevertheless, it is worth noting that the flax based laminates (Flax-T and F-Hybrid-T samples) are characterized by a feeble higher strength and stiffness compared to jute based ones (Jute-T and J-Hybrid-T samples).

These results indicate that the hybridization does not bring benefits if the vegetable fibers are treated in sodium bicarbonate solution. As previously stated, this NaHCO_3_ treatment implied a reduction of the adhesion interaction of the jute fibers with the epoxy resin. Vice versa, there is an increase in the adhesive properties of the initially flexible flax fibers. This involve a softening of the jute based laminates and a stiffening effect on the laminates with the predominant flax laminae. 

This behavior can be ascribed to the different interaction of the two natural fibers with the sodium bicarbonate treatment. Flax fibers are mainly constituted by cellulose (i.e., about 90%) and a low content of lignin (i.e., up to 4%) [51]. Conversely, jute fibers show cellulose and lignin contents equal to about 70% and 25%, respectively. It is known that the sodium bicarbonate treatment is able to remove impurities, such as wax and fatty substances, from the fiber surface [21] as well as to reduce the amounts of hemicellulose and lignin [18]. Therefore, NaHCO_3_ treatment could induce a softening effect on jute fibers, due to their higher amount of lignin in comparison to flax fibers. With regard to flax fibers, the proposed treatment leads just to the removal of the impurities in the fiber skin but it is not able to chemically interact within the fiber core due both to the lower lignin content and to a more compact structure, in comparison to jute fibers [48]. 

Further studies aimed to deeper investigate the phenomena that lead to this different response of jute and flax to the sodium bicarbonate treatment will be carried out in future studies, with the aim to highlight the superficial interaction of natural fibers with the NaHCO_3_ alkaline solution and the related modifications induced by this interaction.

### 3.2. Dynamic Mechanical Thermal Analysis

#### 3.2.1. Effect of Sodium Bicarbonate Treatment

**Figure 6:**
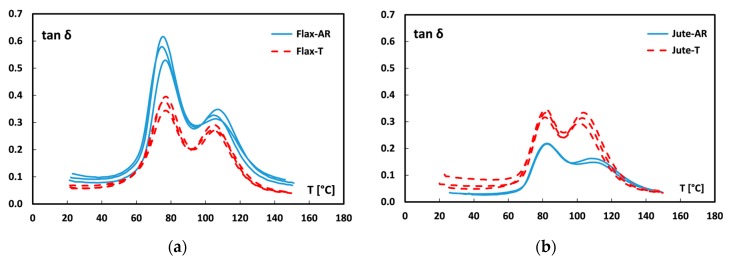
Damping factor curves of (**a**) flax and (**b**) jute monolithic laminates.

The trends of tan δ (i.e., damping factor) versus temperature for all the fiber monotype laminates are shown in Figure 6. As is widely known [52,53,54], the curve shape of damping factor is greatly affected by the fiber–matrix adhesion since the loss to storage modulus ratio (E″/E′) is strictly depend on dissipative phenomena. In particular, the tan δ modification can be associated both to shear stresses concentration at the fiber/matrix interface and to viscoelastic energy dissipation in the bulk of the matrix [15]. Therefore, a high damping value takes place in soft fiber–matrix interfaces. Conversely, an effective adhesion between the composite constituents implies a stiffer fiber-matrix interface, thus leading to the decrease of the damping factor [52].

By observing Figure 6, it is worth noting that all the curves present two different peaks. As described in previous papers [20,55,56], the first peak is related to the glass transition temperature of the epoxy matrix whereas the second peak at higher temperatures can be ascribed to micro-mechanical transition due to the presence of an immobilized polymer layer between fiber and matrix: i.e., fiber–matrix interphase [57]. 

By comparing untreated laminates (blue curves in Figure 6), it is possible to note that both peaks are found at higher temperatures for jute laminates than flax ones (82 ± 0.1 °C vs. 75.4 ± 1.5 °C and 108.3 ± 1.0 °C vs. 105.6 ± 1.3 °C for the first and second peak, respectively). Furthermore, jute laminates showed lower magnitude of both peaks. Since a weak fiber-matrix adhesion leads to higher values of tan δ [52], while a stronger interface limits the mobility of the polymer chains, thus reducing the damping, these results confirm that, without any treatment, jute fabrics present better adhesion compatibility to the epoxy resin, in comparison to flax fabrics.

Regarding to the effect of the sodium bicarbonate treatment, the shift observed in the damping factor curves are in agreement with the results of the quasi-static flexural characterization. In particular, Figure 6a shows that by soaking flax fabrics in sodium bicarbonate solution, the temperature of both peaks remains almost constant whereas the peaks magnitude noticeably decreases (i.e., 0.574 ± 0.043 vs. 0.372 ± 0.026 and 0.329 ± 0.017 vs. 0.279 ± 0.012, respectively), thus confirming the beneficial effect of the proposed treatment on the adhesion between flax fiber and epoxy resin. Vice versa, Jute-T laminates show both damping peaks at the same temperatures but with greater heights in comparison to laminates reinforced with raw jute fabrics. This experimental evidence confirms that a slight worsening of the adhesion between jute fibers and the epoxy resin is achieved after the sodium bicarbonate treatment.

#### 3.2.2. Effect of Hybridization

**Figure 7:**
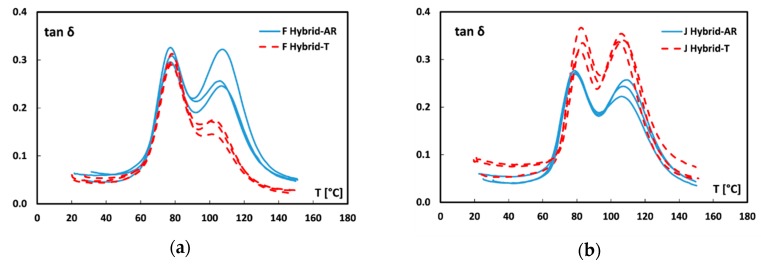
Damping factor curves of (**a**) flax hybrid and (**b**) jute hybrid laminates.

Figure 7 shows the tan δ trends at increasing temperature for all hybrid laminates. Analogously to dynamic mechanical thermal analysis (DMTA) results obtained for fiber monotype laminates (Figure 6), it is possible to note a reduction of the damping factor in F-Hybrid-T laminates and an increase in J-Hybrid-T laminates.

In particular, for flax based hybrid laminates (Figure 7a) the sodium bicarbonate treatment induces a reduction mainly of the secondary peak at high temperature. This indicates that an enhancement of interfacial adhesion occurred. On the other hand, the quite constant magnitude of the main peak at low temperature, related to T_g_ of the matrix, indicates that no modification in chain mobility of the epoxy matrix can be identified. A more evident modification of the dynamic mechanical behavior takes place on jute based hybrid laminates (Figure 7b) due to the fiber treatment. In this case, the sodium bicarbonate treatment of the fibers implies an increase of both tan δ peaks. This behavior is associated, on the one hand, with the reduction of the interface properties of jute fibers (i.e., peak at about 110 °C), which plays an important role in this staking sequence. At the same time, there is an increase in magnitude of the main peak at about 80 °C. This could be due to a less effective cross-linking of the matrix induced by the low chemical interaction between jute fibers and matrix, which may have induced a larger chain mobility in the bulk of the matrix itself [52,58].

The dynamic mechanical thermal characterization confirms the suitable and reliable effect of the stacking sequence hybridization of coupled flax-jute fibers. Nevertheless, the sodium bicarbonate treatment on fibers significantly hinders the beneficial effects of hybridization on the lignocellulosic composite laminates thus limiting its efficiency on static and dynamic mechanical performances.

### 3.3. Impact Tests

It is widely known that, by acquiring data concerning the impact resistance of composite structures, important information about fiber-matrix adhesion and properties of matrix and fiber can be obtained [40]. In particular, when a fiber-reinforced material undergoes a sudden load, the impact energy is dissipated by the combination of fiber fractures, fiber pull-outs, and matrix deformations [59].

#### 3.3.1. Effect of Sodium Bicarbonate Treatment 

**Figure 8:**
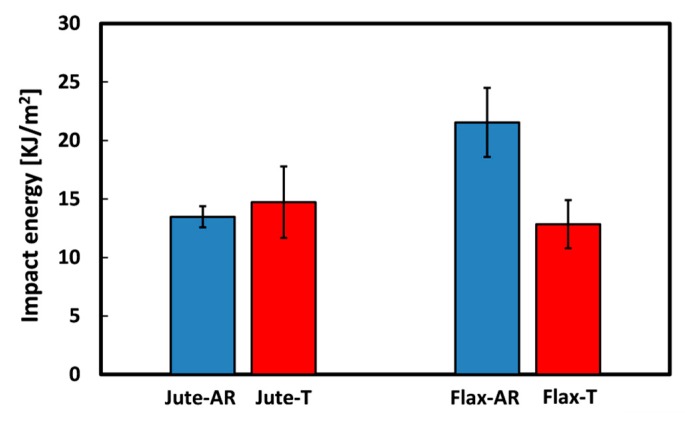
Impact energy of monolithic laminates.

The average values of the impact energy per unit cross-sectional area for all the fiber monotype laminates are shown in Figure 8. First of all, it is worth considering that the impact results revealed a different behavior between the untreated natural fiber-based laminates. In particular, the impact energy of unaged Flax-AR laminates is 46% higher than jute ones. This result is in accordance with those achieved from the quasi-static characterization: i.e., the higher energy absorption capability shown by flax laminates can be ascribed to the greater toughness of flax dry fabrics in comparison to their counterparts (Figure 2). Regarding to the effect of the sodium bicarbonate treatment, it is shown that the impact energy of jute laminates remains almost constant whereas a relevant decrease occurred for flax laminates (i.e., −40%). This experimental evidence confirms the beneficial effect of the NaHCO_3_ treatment on the adhesion compatibility between flax fibers and epoxy matrix. Indeed, other authors showed how the improvement of the compatibility between flax fibers and epoxy matrix leads to the reduction of the composites impact energy [60]. 

Table 3 summarizes the impact properties of all the resulting laminates. The best impact energy of the Flax-AR laminates can be ascribed to a quite double deflection at breaks, compared to other samples. The decrement of the impact energy of flax composites after the sodium bicarbonate treatment is mainly due to the reduced deflection at break experienced by Flax-T laminates (i.e., 4.2%), noticeable lower than that of Flax-AR laminates (i.e., 7.7%). Furthermore, a peak load increase is shown after the NaHCO_3_ treatment (i.e., 180.6 N vs. 130.5 N), which evidences the improvement of fiber-matrix adhesion due to the soaking of flax fabrics in the sodium bicarbonate solution. Vice versa, the quite constant impact energy of jute laminates can be explained considering that the eco-friendly treatment leads to dissimilar effects on the variation of the peak load and of the deflection at break. While the peak load decreases after the sodium bicarbonate treatment (i.e., from 284.8 N to 223.6 N), the deflection at break of Jute-T laminates (i.e., 3.7%) results greater than that of untreated jute ones (i.e., 2.9%). These results are in accordance with those of quasi-static and dynamic mechanical thermal tests, confirming the slight detrimental effect of the proposed treatment on the adhesion between jute fibers and the epoxy resin.

By analyzing the effect of the sodium bicarbonate treatment on hybrid configurations, it is worth noting that the impact energy of J-Hybrid laminates slightly increases after the treatment (i.e., from 13.79 kJ/m^2^ to 15.18 kJ/m^2^) while F-Hybrid-T laminates show lower impact energy (i.e., 14.72 kJ/m^2^) than F-Hybrid AR ones (i.e., 18.77 kJ/m^2^). These results can be explained considering that, as already stated, the NaHCO_3_ treatment certainly improved the fiber-matrix compatibility for flax fibers whereas a slight worsening effect is found for jute fibers. Hence, the hybrid stacking sequence having more flax fabrics (i.e., F-Hybrid laminates) experiences a global decrement of the impact energy while J-Hybrid laminates decrease their impact energy after the fiber treatment.

#### 3.3.2. Effect of Hybridization 

**Figure 9:**
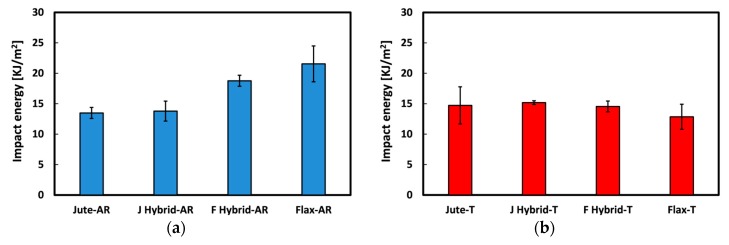
Impact energy at varying stacking sequence of (**a**) untreated and (**b**) treated laminates.

The impact energy per unit cross-sectional area at varying the stacking sequence for untreated and treated natural fiber composite laminates is reported in Figure 9.

The hybridization has a relevant effect on the impact energy magnitude of untreated composite laminates (Figure 9a). A progressive increase of the impact energy at increasing flax content in the stacking sequence configuration can be identified. The impact performances of hybrid composites are intermediate to the respective monolithic composite ones. However, the laminate with the external flax fabrics, characterized by a greater resilience with respect to jute fabrics (identifiable also comparing Jute-AR and Flax-AR samples), shows a significant increase in impact energy compared to the hybrid laminate with the complementary stacking sequence. Indeed, F-Hybrid-AR laminates exhibit an impact energy about 36% higher than J-Hybrid-AR laminates (Table 3). This implies that this stacking sequence configuration, for untreated natural fiber laminates, allows to obtain a mechanical performance compromise between these vegetable fibers by enhancing their strength properties (i.e., jute fibers) and flexibility (i.e., flax fibers).

Vice versa, by analyzing the impact energy trend for hybrid laminates reinforced with treated fibers (Figure 9b) an evident uniformity of data is observed: i.e., no noticeable differences can be identified. The soaking of both vegetable fibers in sodium bicarbonate solution homogenized the performances of the fibers inducing a dissimilar effect on jute and flax fibers adhesion. Consequently, in this case the hybridization choice in the composite design does not have any beneficial effect on the final performance of the hybrid laminates. These considerations are congruent to bending observations, where quite compatible results concerning the performance efficiency of hybridized composites were achieved. 

Overall, it can be concluded that the sodium bicarbonate treatment influences the impact performances of the monolithic laminates whereas no noticeable changes are found for the hybrid configurations. Consequently, it is possible to tailor the impact properties of the resulting laminates by varying the stacking sequence just using untreated fabric as reinforcement. Vice versa, the impact properties of the laminates reinforced with treated fibers are found to be quite constant, regardless the stacking sequence.

### 3.4. Morphological Analysis

**Figure 10:**
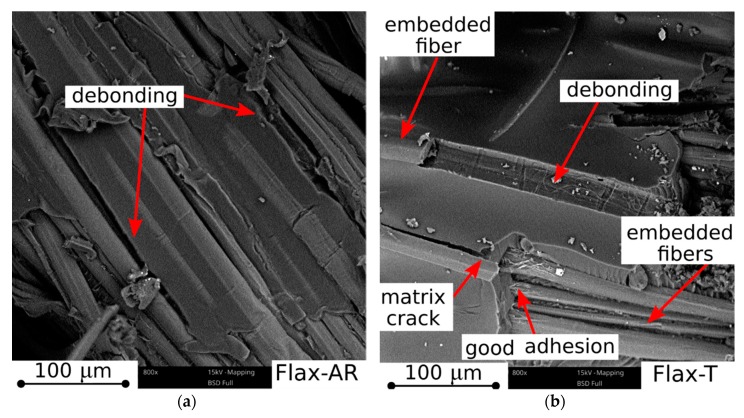

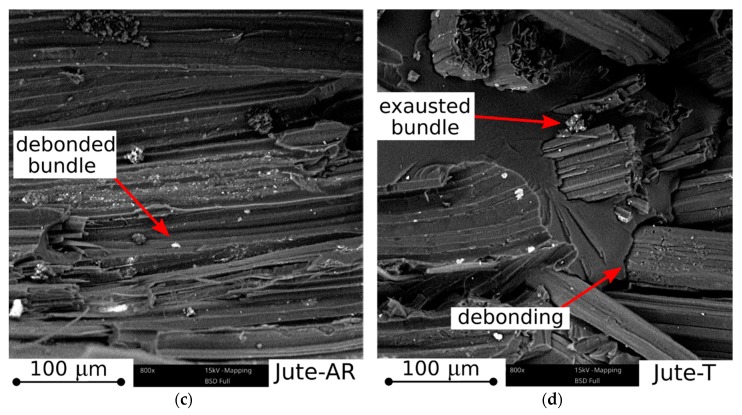
SEM micrographs of impact fracture surfaces of untreated and treated jute and flax reinforced composites: (**a**) Flax-AR, (**b**) Flax-T, (**c**) Jute-AR, and (**d**) Jute-T samples.

The morphology of the impact-fractured surfaces of untreated and treated jute and flax reinforced composites is shown in Figure 10. 

The Flax-AR sample (Figure 10a) is characterized by large and extended cracks, due to debonding, triggered and propagated at the fiber/matrix interface: i.e., long gaps can be detected at the fiber-matrix interface without the presence of the epoxy matrix sticking on the fiber surface. Conversely, the untreated jute sample (i.e., Jute-AR), shown in Figure 10c, exhibits a higher level of adhesion, evidencing a fracture surface without evident cracks. The structure is compact and some surface grooves due to debonding of fiber bundles can be observed. This morphology suggests a suitable adhesion of as received jute fabrics with the epoxy resin, whereas in the Flax-AR sample a lower fiber-matrix compatibility can be considered as responsible of the lower mechanical performances observed for this batch. Furthermore, by analyzing the fracture surfaces of epoxy-based laminates reinforced with treated fabrics (i.e., Flax-T and Jute T shown in Figure 10b,d, respectively), useful considerations concerning the effect of the NaHCO_3_ treatment on fiber/matrix adhesion can be argued. In particular, few debonding regions can be identified in the fracture surface of Flax-T. At the same time, the interface area between fiber and matrix does not show evident detachment zone. Occasionally matrix cracks propagation takes place. This fracture morphology is compatible with previously discussed mechanical results. Due to the sodium bicarbonate treatment, flax fibers improve their adhesion with the epoxy matrix, thus leading to a consequent strengthening and stiffening of the resulting composites. The presence of matrix cracks clearly evidences both that the stress transfer between fiber and matrix is effective and that the limiting factor in the laminate resistance is not due to fiber debonding, but it can be related to the crack propagation within the matrix bulk. Finally, by analyzing the fracture surface of the Jute-T sample, there is evidence of the detrimental effect of the sodium bicarbonate treatment on the fiber-matrix adhesion compatibility. Indeed, the fiber bundle is often exhausted and unraveled limiting an effective interaction with the epoxy matrix. This morphology results in a higher risk of debonding and consequent premature failure of the laminate at lower stress levels compared to untreated laminate. 

## 4. Conclusions

All the experimental tests clearly show that, before the sodium bicarbonate treatment, jute fibers exhibited a suitable adhesion with the epoxy resin whereas lower compatibility was experienced by flax fibers. Furthermore, this treatment allowed to improve the epoxy resin-flax fibers adhesion while a slight worsening effect was observed for jute fabrics. Morphological investigation of the fracture surfaces by SEM clearly supported these statements.

As a consequence, the main results can be summarized as follows:

- Jute-AR laminates, showing a predominantly elastic behavior with a brittle and catastrophic fracture, evidenced higher strength and stiffness but lower strain at maximum stress than Flax-AR laminates, characterized by a mainly elasto-plastic behavior. These results can be ascribed both to the mechanical behavior of dry fabrics and to the fiber adhesion with the epoxy resin used as matrix;
- The NaHCO_3_ treatment led to noticeable improvements of the quasi-static properties of flax reinforced composites whereas slight decrements of strength and modulus were exhibited by jute composites. This different behavior was also shown by dynamic-mechanical-thermal analysis and impact characterization;
- The hybridization had a relevant effect on the impact behavior of untreated composite laminates: i.e., a progressive increase of the impact energy at increasing flax content in the stacking sequence configuration was identified. Furthermore, hybrid laminates with external flax fabrics (i.e., F-Hybrid-AR) showed better quasi-static mechanical behavior than complementary stacking sequence with external jute laminas (i.e., J-Hybrid-AR); and
- The coupled action of hybridization and NaHCO_3_ treatment does not lead to a beneficial and reliable effect on the mechanical response of the investigated composite laminates (i.e., F-hybrid-T and J-Hybrid-T).

## Figures and Tables

**Table 1:** List of manufactured composite laminates.

Code	Stacking Sequence ^1^	Fabrics	Thickness (mm)	Fiber Content (%)	*ρ_e_* (g/cm^3^)	*ρ_t_* (g/cm^3^)	Void Content (%)
Flax-AR	[F]_5_	as received	3.38 ± 0.03	0.316	1.234	1.287	4.12
Flax-T	[F]_5_	treated	3.71 ± 0.04	0.273	1.279	1.239	3.17
Jute-AR	[J]_5_	as received	4.72 ± 0.09	0.326	1.301	1.237	4.91
Jute-T	[J]_5_	treated	5.65 ± 0.05	0.258	1.282	1.225	4.48
F-Hybrid-AR	[F/J/F/J/F]	as received	3.96 ± 0.03	0.296	1.266	1.211	4.31
F-Hybrid-T	[F/J/F/J/F]	treated	4.39 ± 0.11	0.255	1.260	1.208	4.11
J-Hybrid-AR	[J/F/J/F/J]	as received	4.19 ± 0.05	0.338	1.274	1.213	4.77
J-Hybrid-T	[J/F/J/F/J]	treated	4.90 ± 0.17	0.279	1.264	1.207	4.48

^1^ F = twill weave flax fabric; J = twill weave woven jute fabric.

**Table 2:** Flexural properties of monolithic laminates.

CODE	Flexural Strength (MPa)	Flexural Modulus (GPa)	Strain at Maximum Stress (%)
Flax-AR	73.7 ± 6.0	3.75 ± 0.47	5.2 ± 0.2
Flax-T	103.5 ± 7.3	5.48 ± 0.23	3.0 ± 0.3
Jute-AR	87.6 ± 3.7	4.36 ± 0.19	3.0 ± 0.1
Jute-T	80.8 ± 3.7	4.42 ± 0.12	2.4 ± 0.1

**Table 3:** Impact properties of laminates.

CODE	Impact Energy (kJ/m^2^)	Peak Load (N)	Deflection at Break (mm)
Flax-AR	21.54 ± 2.95	130.5 ± 11.0	7.7 ± 0.5
Flax-T	12.85 ± 2.06	180.6 ± 13.1	4.2 ± 0.3
Jute-AR	13.48 ± 0.90	284.8 ± 25.7	2.9 ± 0.3
Jute-T	14.72 ± 3.05	223.6 ± 20.7	3.7 ± 0.3
F-Hybrid-AR	18.77 ± 0.90	186.9 ± 11.7	5.4 ± 0.4
F-Hybrid-T	14.54 ± 0.90	225.5 ± 11.2	3.7 ± 0.2
J-Hybrid-AR	13.79 ± 1.64	237.8 ± 12.7	3.5 ± 0.3
J-Hybrid-T	15.18 ± 0.31	178.0 ± 5.8	4.2 ± 0.3

## References

[1] Fortea-Verdejo M., Bumbaris E., Burgstaller C., Bismarck A., Lee K.Y. (2017). Plant fibre-reinforced polymers: Where do we stand in terms of tensile properties?. Int. Mater. Rev..

[2] Sarasini F., Fiore V. (2018). A systematic literature review on less common natural fibres and their biocomposites. J. Clean. Prod..

[3] Summerscales J., Dissanayake N., Virk A., Hall W. (2010). A review of bast fibres and their composites. Part 2—composites. Compos. Part A Appl. Sci. Manuf..

[4] Mohanty A.K., Vivekanandhan S., Pin J.-M., Misra M. (2018). Composites from renewable and sustainable resources: Challenges and innovations. Science.

[5] Davoodi M.M., Sapuan S.M., Ahmad D., Ali A., Khalina A., Jonoobi M. (2010). Mechanical properties of hybrid kenaf/glass reinforced epoxy composite for passenger car bumper beam. Mater. Des..

[6] Kabir M.M., Wang H., Lau K.T., Cardona F. (2012). Chemical treatments on plant-based natural fibre reinforced polymer composites: An overview. Compos. Part B Eng..

[7] Dittenber D.B., Gangarao H.V.S. (2012). Critical review of recent publications on use of natural composites in infrastructure. Compos. Part A Appl. Sci. Manuf..

[8] Scalici T., Fiore V., Valenza A. (2016). Effect of plasma treatment on the properties of Arundo Donax L. leaf fibres and its bio-based epoxy composites: A preliminary study. Compos. Part B Eng..

[9] Rao J., Bao L., Wang B., Fan M., Feo L. (2018). Plasma surface modification and bonding enhancement for bamboo composites. Compos. Part B Eng..

[10] Ragoubi M., Bienaimé D., Molina S., George B., Merlin A. (2010). Impact of corona treated hemp fibres onto mechanical properties of polypropylene composites made thereof. Ind. Crops Prod..

[11] Li X., Tabil L.G., Panigrahi S. (2007). Chemical treatments of natural fiber for use in natural fiber-reinforced composites: A review. J. Polym. Environ..

[12] Chandrasekar M., Ishak M.R., Sapuan S.M., Leman Z., Jawaid M. (2017). A review on the characterisation of natural fibres and their composites after alkali treatment and water absorption. Plast. Rubber Compos..

[13] Cai M., Takagi H., Nakagaito A.N., Li Y., Waterhouse G.I.N. (2016). Effect of alkali treatment on interfacial bonding in abaca fiber-reinforced composites. Compos. Part A Appl. Sci. Manuf..

[14] Yan L., Chouw N., Yuan X. (2012). Improving the mechanical properties of natural fibre fabric reinforced epoxy composites by alkali treatment. J. Reinf. Plast. Compos..

[15] Fiore V., Di Bella G., Valenza A. (2015). The effect of alkaline treatment on mechanical properties of kenaf fibers and their epoxy composites. Compos. Part B Eng..

[16] Singh G.P., Madiwale P.V., Jagtap R.N., Adivarekar R.V. (2014). Extraction of fibers from saccharum munja grass and its application in composites. J. Appl. Polym. Sci..

[17] Fiore V., Scalici T., Nicoletti F., Vitale G., Prestipino M., Valenza A. (2016). A new eco-friendly chemical treatment of natural fibres: Effect of sodium bicarbonate on properties of sisal fibre and its epoxy composites. Compos. Part B Eng..

[18] Santos J.C., Siqueira R.L., Vieira L.M.G., Freire R.T.S., Mano V., Panzera T.H. (2018). Effects of sodium carbonate on the performance of epoxy and polyester coir-reinforced composites. Polym. Test..

[19] dos Santos J.C., de Oliveira L.Á., Gomes Vieira L.M., Mano V., Freire R.T.S., Panzera T.H. (2019). Eco-friendly sodium bicarbonate treatment and its effect on epoxy and polyester coir fibre composites. Constr. Build. Mater..

[20] Fiore V., Scalici T., Valenza A. (2018). Effect of sodium bicarbonate treatment on mechanical properties of flax-reinforced epoxy composite materials. J. Compos. Mater..

[21] Chaitanya S., Singh I. (2017). Sisal fiber-reinforced green composites: Effect of ecofriendly fiber treatment. Polym. Compos..

[22] Selver E., Ucar N., Gulmez T. (2018). Effect of stacking sequence on tensile, flexural and thermomechanical properties of hybrid flax/glass and jute/glass thermoset composites. J. Ind. Text..

[23] Kumar S., Gangil B., Prasad L., Kumar Patel V. (2017). A Review on mechanical behaviour of bast-glass fibre based hybrid polymer composites. Mater. Today Proc..

[24] Sanjay M.R., Arpitha G.R., Yogesha B. (2015). Sudy on mechanical properties of natural—glass fibre reinforced polymer hybrid composites: A review. Mater. Today Proc..

[25] Fiore V., Calabrese L., Scalici T., Bruzzaniti P., Valenza A. (2018). Bearing strength and failure behavior of pinned hybrid glass-flax composite laminates. Polym. Test..

[26] Sarasini F., Tirillò J., D’Altilia S., Valente T., Santulli C., Touchard F., Chocinski-Arnault L., Mellier D., Lampani L., Gaudenzi P. (2016). Damage tolerance of carbon/flax hybrid composites subjected to low velocity impact. Compos. Part B Eng..

[27] Bagheri Z.S., El Sawi I., Schemitsch E.H., Zdero R., Bougherara H. (2013). Biomechanical properties of an advanced new carbon/flax/epoxy composite material for bone plate applications. J. Mech. Behav. Biomed. Mater..

[28] Fiore V., Valenza A., Di Bella G. (2012). Mechanical behavior of carbon/flax hybrid composites for structural applications. J. Compos. Mater..

[29] Cicala G., Pergolizzi E., Piscopo F., Carbone D., Recca G. (2018). Hybrid composites manufactured by resin infusion with a fully recyclable bioepoxy resin. Compos. Part B Eng..

[30] Dhakal H.N., Zhang Z.Y., Guthrie R., MacMullen J., Bennett N. (2013). Development of flax/carbon fibre hybrid composites for enhanced properties. Carbohydr. Polym..

[31] Longana M., Ondra V., Yu H., Potter K., Hamerton I. (2018). Reclaimed Carbon and Flax Fibre Composites: Manufacturing and Mechanical Properties. Recycling.

[32] Longana M.L., Yu H., Aryal P., Potter K.D. The High Performance Discontinuous Fibre (HiPerDiF) Method for Carbon-Flax Hybrid Composites Manufacturing. Proceedings of the 21st International Conference on Composite Materials.

[33] Bhanupratap R., Chittappa H.C. (2018). Morphological Study of the Flexural Behaviour of Nanoclay Filled Jute/Kevlar Reinforced Epoxy Hybrid Composite. IOP Conf. Ser. Mater. Sci. Eng..

[34] Audibert C., Andreani A.S., Lainé É., Grandidier J.C. (2018). Mechanical characterization and damage mechanism of a new flax-Kevlar hybrid/epoxy composite. Compos. Struct..

[35] Almansour F.A., Dhakal H.N., Zhang Z.Y. (2017). Effect of water absorption on Mode I interlaminar fracture toughness of flax/basalt reinforced vinyl ester hybrid composites. Compos. Struct..

[36] Suresh Kumar C., Arumugam V., Dhakal H.N., John R. (2015). Effect of temperature and hybridisation on the low velocity impact behavior of hemp-basalt/epoxy composites. Compos. Struct..

[37] Almansour F.A., Dhakal H.N., Zhang Z.Y. (2018). Investigation into Mode II interlaminar fracture toughness characteristics of flax/basalt reinforced vinyl ester hybrid composites. Compos. Sci. Technol..

[38] Fiore V., Scalici T., Calabrese L., Valenza A., Proverbio E. (2016). Effect of external basalt layers on durability behaviour of flax reinforced composites. Compos. Part B Eng..

[39] Fiore V., Scalici T., Sarasini F., Tirilló J., Calabrese L. (2017). Salt-fog spray aging of jute-basalt reinforced hybrid structures: Flexural and low velocity impact response. Compos. Part B Eng..

[40] Fiore V., Scalici T., Badagliacco D., Enea D., Alaimo G., Valenza A. (2017). Aging resistance of bio-epoxy jute-basalt hybrid composites as novel multilayer structures for cladding. Compos. Struct..

[41] Karaduman Y., Onal L., Rawal A. (2015). Effect of stacking sequence on mechanical properties of hybrid flax/jute fibers reinforced thermoplastic composites. Polym. Compos..

[42] Mohan K., Rajmohan T. (2017). Fabrication and characterization of MWCNT filled hybrid natural fiber composites. J. Nat. Fibers.

[43] Chaudhary V., Bajpai P.K., Maheshwari S. (2018). An investigation on wear and dynamic mechanical behavior of jute/hemp/flax reinforced composites and its hybrids for tribological applications. Fibers Polym..

[44] Rodríguez E., Petrucci R., Puglia D., Kenny J.M., Vázquez A. (2005). Characterization of composites based on natural and glass fibers obtained by vacuum infusion. J. Compos. Mater..

[45] Hammami A., Gebart B.R. (2000). Analysis of the vacuum infusion molding process. Polym. Compos..

[46] Scalici T., Pitarresi G., Badagliacco D., Fiore V., Valenza A. (2016). Mechanical properties of basalt fiber reinforced composites manufactured with different vacuum assisted impregnation techniques. Compos. Part B Eng..

[47] Gassan J., Bledzki A.K. (1999). Possibilities for improving the mechanical properties of jute/epoxy composites by alkali treatment of fibres. Compos. Sci. Technol..

[48] Hamad S.F., Stehling N., Holland C., Foreman J.P., Rodenburg C. (2017). Low-Voltage SEM of natural plant fibers: Microstructure properties (surface and cross-section) and their link to the tensile properties. Procedia Eng..

[49] Spārniņš E., Nyström B., Andersons J. (2012). Interfacial shear strength of flax fibers in thermoset resins evaluated via tensile tests of UD composites. Int. J. Adhes. Adhes..

[50] Charlet K., Béakou A. (2011). Mechanical properties of interfaces within a flax bundle—Part I: Experimental analysis. Int. J. Adhes. Adhes..

[51] Mustata F.S.C., Mustata A. (2014). Dielectric behaviour of some woven fabrics on the basis of natural cellulosic fibers. Adv. Mater. Sci. Eng..

[52] Shanmugam D., Thiruchitrambalam M. (2013). Static and dynamic mechanical properties of alkali treated unidirectional continuous Palmyra Palm Leaf Stalk Fiber/jute fiber reinforced hybrid polyester composites. Mater. Des..

[53] Martínez-Hernández A.L., Velasco-Santos C., de-Icaza M., Castaño V.M. (2007). Dynamical–mechanical and thermal analysis of polymeric composites reinforced with keratin biofibers from chicken feathers. Compos. Part B Eng..

[54] Jawaid M., Abdul Khalil H.P.S., Hassan A., Dungani R., Hadiyane A. (2013). Effect of jute fibre loading on tensile and dynamic mechanical properties of oil palm epoxy composites. Compos. Part B Eng..

[55] Pothan L.A., George C.N., John M.J., Thomas S. (2010). Dynamic mechanical and dielectric behavior of banana-glass hybrid fiber reinforced polyester composites. J. Reinf. Plast. Compos..

[56] Fiore V., Calabrese L., Di Bella G., Scalici T., Galtieri G., Valenza A., Proverbio E. (2016). Effects of aging in salt spray conditions on flax and flax/basalt reinforced composites: Wettability and dynamic mechanical properties. Compos. Part B Eng..

[57] Calabrese L., Fiore V., Scalici T., Valenza A. (2019). Experimental assessment of the improved properties during aging of flax/glass hybrid composite laminates for marine applications. J. Appl. Polym. Sci..

[58] Aziz S.H., Ansell M.P., Clarke S.J., Panteny S.R. (2005). Modified polyester resins for natural fibre composites. Compos. Sci. Technol..

[59] Wambua P., Ivens J., Verpoest I. (2003). Natural fibres: Can they replace glass in fibre reinforced plastics?. Compos. Sci. Technol..

[60] Yan L. (2012). Effect of alkali treatment on vibration characteristics and mechanical properties of natural fabric reinforced composites. J. Reinf. Plast. Compos..

